# Biological effects of paenilamicin, a secondary metabolite antibiotic produced by the honey bee pathogenic bacterium *Paenibacillus larvae*

**DOI:** 10.1002/mbo3.195

**Published:** 2014-07-16

**Authors:** Eva Garcia-Gonzalez, Sebastian Müller, Gillian Hertlein, Nina Heid, Roderich D Süssmuth, Elke Genersch

**Affiliations:** 1 Department of Molecular Microbiology and Bee Diseases, Institute for Bee ResearchFriedrich-Engels-Str. 32, 16540, Hohen Neuendorf, Germany; 2 Institut für Chemie, Technische Universität Berlin10623, Berlin, Germany; 3 Institute of Microbiology and Epizootics, Freie Universität BerlinRobert-von-Ostertag-Str. 7-13, 14163, Berlin, Germany

**Keywords:** Antibacterial activity, antifungal activity, cytotoxic activity, nonribosomal peptide-polyketide hybrid, *Paenibacillus larvae*, paenilamicin

## Abstract

*Paenibacillus larvae* is the etiological agent of American Foulbrood (AFB) a world-wide distributed devastating disease of the honey bee brood. Previous comparative genome analysis and more recently, the elucidation of the bacterial genome, provided evidence that this bacterium harbors putative functional nonribosomal peptide synthetases (NRPSs) and polyketide synthases (PKSs) and therefore, might produce nonribosomal peptides (NRPs) and polyketides (PKs). Such biosynthesis products have been shown to display a wide-range of biological activities such as antibacterial, antifungal or cytotoxic activity. Herein we present an *in silico* analysis of the first NRPS/PKS hybrid of *P. larvae* and we show the involvement of this cluster in the production of a compound named paenilamicin (Pam). For the characterization of its *in vitro* and *in vivo* bioactivity, a knock-out mutant strain lacking the production of Pam was constructed and subsequently compared to wild-type species. This led to the identification of Pam by mass spectrometry. Purified Pam-fractions showed not only antibacterial but also antifungal and cytotoxic activities. The latter suggested a direct effect of Pam on honey bee larval death which could, however, not be corroborated in laboratory infection assays. Bee larvae infected with the non-producing Pam strain showed no decrease in larval mortality, but a delay in the onset of larval death. We propose that Pam, although not essential for larval mortality, is a virulence factor of *P. larvae* influencing the time course of disease. These findings are not only of significance in elucidating and understanding host–pathogen interactions but also within the context of the quest for new compounds with antibiotic activity for drug development.

## Introduction

Insect pathogenic bacteria have only recently been recognized as a rich but poorly explored source of structurally diverse secondary metabolites. Nonribosomal peptides (NRPs) and polyketides (PKs) produced by entomopathogens have been shown to have important biological roles during pathogenesis such as antagonism of microbial competitors, motility, and contribution to virulence. In addition, some of them might even have a future as pharmaceuticals (reviewed in Bode 2009). The emergence of whole genome-sequencing projects and improved genome mining techniques paved the way for the identification of new NRP synthetase (NRPS) and PK synthase (PKS) gene clusters. To date, known bacterial entomopathogens producing secondary metabolites are for instance *Bacillus thuringiensis* (Broderick et al. 2000), *Pseudomonas entomophila* (Vallet-Gely et al. 2010), *Serratia marcescens* (Li et al. 2005), and *Xenorhabdus* ssp. (Reimer et al. 2009). Recently, *Paenibacillus larvae* has been added to the list of secondary metabolite producing bacterial entomopathogens (Fünfhaus et al. 2009). Comparative genomics and whole genome comparison within the species *P. larvae* revealed the existence of several NRPS and NRPS/PKS gene clusters (Fünfhaus et al. 2009; Djukic et al. 2014; Schild et al. 2014) including an NRPS gene cluster leading to the production of a novel nonribosomal peptide antibiotic, sevadicin (Garcia-Gonzalez et al. 2014).

*Paenibacillus larvae* is the etiological agent of AFB, a serious disease of the European honey bee (*Apis mellifera*) larvae (Genersch et al. 2006). AFB is the most contagious and destructive bacterial disease affecting honey bees. Although only larvae are susceptible to and inevitably die from the disease, AFB is able to kill entire colonies. Hence, AFB is a notifiable epizootic in most countries and in the case of an outbreak, burning of diseased colonies and contaminated hive material is usually considered the only workable control measure. As a result, AFB leads to considerable economic losses in apiculture. In addition, colony losses due to AFB also compromise agricultural and natural ecosystems because of the indispensable role of honey bees as pollinators of many fruit, crops, and wild flowers.

Despite the enormous impact of this disease, molecular mechanisms of infection are not fully understood. To date, we know that four different genotypes within the species *P. larvae* exist named ERIC I to ERIC IV based on the primers used for differentiation (Genersch et al. 2006). These genotypes not only differ in virulence (Genersch et al. 2005; Rauch et al. 2009) but also in their genomic makeup (Fünfhaus et al. 2009; Djukic et al. 2014). All genotypes share the general steps in pathogenesis: Honey bee larvae become infected by ingesting spores, which germinate in the midgut; vegetative *P. larvae* then massively proliferate in the midgut, breach the peritrophic membrane and the epithelium, and finally decompose the larva to a ropy mass (Yue et al. 2008; Garcia-Gonzalez and Genersch 2013). Recently, the first virulence factors of *P. larvae* have been elucidated and were proved to be genotype specific: *P. larvae* ERIC I expresses two AB toxins, Plx1 and Plx2, missing in *P. larvae* ERIC II (Fünfhaus et al. 2013) whereas the S-layer protein SplA, involved in bacterial adhesion to the midgut epithelium, was shown to be expressed only in *P. larvae* ERIC II (Fünfhaus and Genersch 2012; Poppinga et al. 2012). The relevance of secondary metabolites like sevadicin (Garcia-Gonzalez et al. 2014) during pathogenesis of *P. larvae* infections still remains elusive.

NRPs and PKs are secondary metabolites synthesized by dedicated complex multimodular biosynthetic machineries termed nonribosomal peptide synthetases (NRPSs) and polyketide synthases (PKSs). Each module consists of at least three domains responsible for a specific catalytic reaction in the incorporation of an amino acid (NRPS) or malonyl coenzyme A (PKS). These modular enzyme machineries sequentially activate and condense specific amino acids or acyl units. The modules of the megaenzymes of the NRPS-type (reviewed in Finking and Marahiel 2004) commonly consist of the following domains: The adenylation (A) domain of an NRPS specifically binds and activates an amino acid by transforming it into an amino acyl adenylate. The thiolation (T) domain covalently binds the activated amino acid to the module via a phosphopantetheinyl arm. The condensation (C) domain catalyzes the formation of a peptide bond between two amino acids of neighbored modules. The terminal module consists of an additional thioesterase (TE) domain which releases the peptide from the NRPS. It may occur that NRPSs contain additional domains for further peptide modification. An important representative of such a domain is the epimerization (E) domain which catalyzes the inversion of the stereocenter of the activated l-amino acid into its d-amino acid enantiomer.

Likewise the modules of type I PKSs consist of at least three core domains (reviewed in Hill 2006): the acyltransferase (AT) domain binds an appropriate acyl unit and transfers it into the acyl carrier protein (ACP) domain. The elongation step is performed by the ketosynthetase (KS) domain which catalyzes the formation of a C–C bond via Claisen condensation with the acyl unit of the next module. Optionally, the present domains for further tailoring of the substrates are ketoreductase (KR) domains that catalyse the reduction of the *β*-keto group to a *β*-hydroxy group. A fully saturated acyl backbone additionally requires the reductive action of dehydratase (DH), and enoyl reductase (ER) domains.

Here, we report on the identification, isolation, and biological characterization of a novel NRP/PK hybrid, named paenilamicin (Pam), produced by *P. larvae*. By means of construction of a gene-inactivation mutant followed by mass spectrometric analysis, we were able to link functionality of the *pam* gene cluster responsible for Pam production and antibiotic activity. Further *in vitro* and *in vivo* assays corroborated the antibacterial, antifungal and cytotoxic activities of this metabolite. Moreover, *in vivo* functional analyses suggested that Pam might act as a putative virulence factor in AFB pathogenesis. The study of antibiotic substances produced by the entomopathogen *P. larvae* is of outstanding interest because of both, the identification of a novel bioactive NRPS/PKS hybrid and the characterization of the biological function of this substance.

## Materials and Methods

### Bacterial strains and culture conditions

Wild-type *P. larvae* bacteria (DSM25430; ERIC II (Genersch et al. 2005)) were cultivated either in MYPGP liquid broth (Dingman and Stahly 1983) or on Columbia sheep blood agar (CSA) plates at 37°C as previously described (Genersch and Otten 2003; Neuendorf et al. 2004). Gene inactivation mutants of *P. larvae* were cultivated on MYPGP-agar plates supplemented with 5 *μ*g/mL chloramphenicol for selection and incubated at 37°C for 2–3 days as previously described (Poppinga et al. 2012).

*Paenibacillus alvei*, *Bacillus subtilis, Bacillus licheniformis, Bacillus megaterium*, and *Saccharomyces cerevisiae* were obtained from the DSMZ (Deutsche Sammlung von Mikroorganismen und Zellkulturen GmbH, Braunschweig, Germany). *Pichia pastoris* yDT39 was a generous gift from Prof. Budisa (Technische Universität, Berlin, Germany) and *Fusarium oxysporum* ETH1536/9 was obtained from the Centraalbureau voor Schimmelcultures (Utrecht, the Netherlands). These strains were used for testing the antibacterial activity of *P. larvae* culture supernatants or purified Pam. All strains were grown in LB (Luria Bertani), MYPGP, YPD or FCM media (broth or agar plates).

*Paenibacillus larvae* spore suspensions were generated by resuspending about 100 colonies in 300 *μ*L brain heart infusion (BHI) broth to inoculate the liquid part of CSA slants. These were incubated at 37°C for 10 days. Spore concentrations were determined by cultivating serial dilutions as already described (Genersch and Otten 2003; Neuendorf et al. 2004).

*Escherichia coli* DH5*α* cells (Invitrogen, Karlsruhe, Germany) transformed with the plasmid pTT_*pam*A (see below) were cultivated in selective LB media (agar and broth) supplemented with 30 *μ*g/mL chloramphenicol. Plasmid DNA was prepared following the manufacturer's protocols (QIAprep Spin Miniprep kit; Qiagen, Hilden, Germany). Concentration and purity of the plasmid preparations were analyzed by photometric analysis (Nanodrop; Peqlab, Erlangen, Germany) and agarose gel electrophoresis.

### *Paenibacillus larvae* cultivation for secondary metabolite production

*Paenibacillus larvae* was cultivated as follows: bacteria were grown on CSA plates at 37°C for 2–3 days. A preculture of 10 mL MYPGP medium was inoculated with a single colony and grown overnight. A 50 mL of MYPGP broth was inoculated with the preculture to reach a final (calculated) OD_600_ of 0.001. This main culture was incubated at 30°C with gentle shaking (80 rpm, Thermoshake; Gerhardt, Bonn, Germany) for 72 h. Cultures were centrifuged for 10 min at 4°C with 3200 *g* and supernatants were stored at −20°C until further use.

### Agar diffusion assay for antibacterial activity

To test the antibiotic activity of *P. larvae* supernatants, *P. alvei*, *B. subtilis, B. licheniformis* and *B. megaterium* were used as bacterial indicator strains and *S. cerevisiae*, *Pichia pastoris*, and *Fusarium oxysporum* as fungal indicator strains. The activity against *P. alvei*, *B. subtilis, B. licheniformis, B. megaterium*, and *S. cerevisiae* was tested as follows. A preculture was grown in 5 mL of the correspondent broth (LB or MYPGP) and cultured overnight at 37°C with gentle shaking. A 10 mL of the correspondent broth was inoculated with 500 *μ*L of this preculture and cultivated at 37°C with gentle shaking for 5 h. Subsequently, the OD_600_ of the cultures (typically between 0.5 and 0.7) was determined. A 40 mL of molten, 37°C warm LB or MYPGP agar was inoculated to reach a final (calculated) OD_600_ of 0.05 and poured on Petri plates, while 20 *μ*L of the test substrate (*P. larvae* supernatants or purified Pam) was dispensed on a filter disk and dried at RT. Subsequently, the disks were carefully placed on the agar. Alternatively, holes of 6 mm in diameter were made into the agar and filled with 40 *μ*L of the tested substance. The plates were incubated at 37°C overnight. Clear zones around the filter disks or the holes were considered indicative of growth inhibition. *P. pastoris* yDT39 was grown in 5 mL YPD medium at 30°C and 200 rpm until reaching OD_600_ of around 0.8–0.9. 200 *μ*L of this culture were plated on YPD agar plates (10 mL per plate). Plates were incubated at 30°C overnight. *F. oxysporum* ETH 1536/9 was grown in 5 mL FCM medium at 27°C and 115 rpm for 48 h. FCM agar plates for bioactivity tests were made the same way as described for *P. pastoris* above. The agar plates were incubated at 27°C for 2–3 days.

### Sequence analysis

The genome sequence of *Paenibacillus larvae* (*P. larvae* DSM25430 (Djukic et al. 2014), NCBI Reference Sequence NC_023134.1) was screened for polyketide synthase (PKS) – and nonribosomal peptide synthetase (NRPS) – coding sequences. This analysis revealed the occurrence of nine genes consisting of five NRPSs, two PKSs, and two NRPS/PKS hybrid genes (NC_023134.1, position 1729923–1788675). Sequence similarity searches were performed using BLASTX and BLASTP at the NCBI website (Altschul et al. 1997). Domain organization, extraction of binding pocket signatures and substrate specificity prediction were analyzed by using *in silico* tools: NRPSpredictor2 (Rausch et al. 2005; Röttig et al. 2011), PKS/NRPS analysis (Bachmann and Ravel 2009), and SBSPKS (Anand et al. 2010).

### Construction of a *P. larvae* gene inactivation mutant (knock-out strain)

The gene *pam*A (Gene ID: 18058116) from the *pam* gene cluster was disrupted in the genome of *P. larvae* DSM25430 via a recently described strategy (Zarschler et al. 2009). Vector pTT_*wsf*A243 (Zarschler et al. 2010) was used for constructing a targetron vector for targeted group II intron insertion at position 1080 from the start codon of *pam*A of the *pam* gene cluster. Retargeting of the LI.LtrB targetron of vector pTT_*wsf*A243 prior to transformation into *P. larvae* was performed following the manufacturer's protocol and essentially as already described for disruption of the *P. larvae* S-layer gene *splA* (Poppinga et al. 2012). In brief, a computer algorithm provided by the manufacturer (http://www.sigma-genosys.com/targetron) identified potential insertion sites for the intron into *pam*A open reading frames and designed suitable PCR primers (Table1) for the modification of the intron RNA. The algorithm predicted a high insertion efficiency for the position 1080 in the gene and designed corresponding PCR primers. These primers were used for modification of the LI.LtrB targetron to generate the new vector pTT_*pam*A, respectively, which were subsequently transformed into *E. coli* DH5*α* cells for plasmid replication and preparation. The targeted sequence for intron insertion was unique inside the DSM25430 genome, assuring the specificity of gene inactivation.

**Table 1:** Primers used in this study.

Primer name	Primer sequences (5′–3′)	Used for
IBS_cltIII_pksA	AAAAAAGCTTATAATTATCCTTATCCGGCGTCGCAGTGCGCCCAGATAGGGTG	creation of *pam*A knock-out
EBS1d_cltIII_pksA	CAGATTGTACAAATGTGGTGATAACAGATAAGTCGTCGCAGGTAACTTACCTTTCTTTGT	
EBS2_cltIII_pksA	TGAACGCAAGTTTCTAATTTCGGTTCCGGATCGATAGAGGAAAGTGTCT	
PKSA_F	GGCACGAGCATGGGTGATCCG	sequencing; verification of *pam*A knock-out
PKSA_R	CGTGGACATTTGTCCCGCCCA	

**Figure 1:**
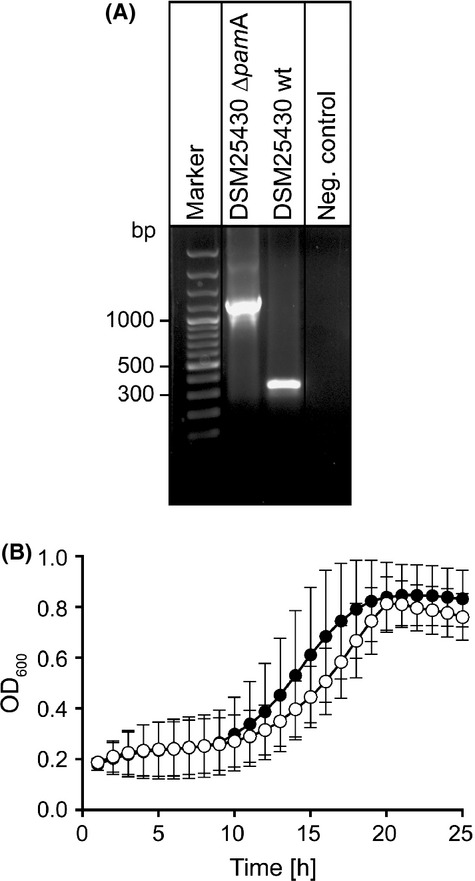
Determination of successful knock-out construction and growth analysis of knockout mutant. (A) PCR analysis of the constructed knock-out mutant DSM25430 Δ*pam*A, revealed successful intron insertion. Primers flanking the insertion sites in the *pam*A gene in the wild-type strain DSM25430 wt, amplified a fragment that was 915 bp smaller than the fragment amplified from DSM25430Δ*pam*A because of intron insertion. (B) Growth of DSM25430 wt and DSM25430 Δ*pam*A was analyzed with three biological and three technical replicates each. No significant difference in growth could be observed (two-way ANOVA, *P *=* *0.607). PCR, polymerase chain reaction.

For generation of a *P. larvae* gene inactivation mutant, electrocompetent *P. larvae* DSM25430 cells (ERIC II) were prepared as described (Murray and Aronstein 2008) and 1 *μ*g of plasmid pTT_*pam*A was transformed by electroporation as recently established (Poppinga and Genersch 2012). Cells were regenerated in MYPGP broth for 16 h, plated on MYPGP-agar containing 5 *μ*g/mL chloramphenicol and incubated for 3 days. Positive clones were identified by PCR analysis using primers flanking the intron insertion positions of the targeted ORF: 948 and 1276 (Table1, Fig.1A), leading to the construction of the strain DSM25430 Δ*pam*A. Germination, growth in liquid broth, and sporulation of *P. larvae* DSM25430 Δ*pam*A was tested and did not significantly differ from the parent wild-type strain (Fig.1B; two-way ANOVA; *P* = 0.607) thus ruling out that the outcome of the exposure bioassays is influenced by unknown side effects of the knock-out strategy on relevant features of the bacteria as also already described for other *P. larvae* knock-out mutants generated with the targetron strategy (Poppinga et al. 2012; Fünfhaus et al. 2013).

### Liquid chromatography-electrospray ionization-mass spectrometry analytics

Polypropylene columns (1 mL) (Qiagen) were filled with Amberlite XAD-16 adsorption beads (Sigma, St. Louis, MO) (1.5 g), and used for subsequent chromatographic fractionation. Thus, prepared columns were then equilibrated by flushing with three column volumes (CVs) of distilled water. Frozen supernatants of *P. larvae* cultures were thawed, and the column was loaded with supernatant. A step gradient was applied using 1 CV of distilled water followed by aqueous methanol of decreasing polarity (1 CV each in 10% steps: 10%, 20%, 30%; etc. 100%). Eluates were tested for bioactivity against *B. megaterium*. Chromatographic fractions from *P. larvae* wild-type showing bioactivity and the corresponding fractions from *P. larvae* gene inactivation mutant were pooled and dried in vacuo. For LC-ESI-MS analytics, the extracts were resolved in 5 mL 10% aqueous acetonitrile + 0.1% formic acid and a portion of 2 *μ*L was injected into the HPLC (high-pressure liquid chromatography) column.

LC-ESI-MS analytics were performed using an Agilent 6410 Triple Quadrupole LC/MS system in MS2-scan mode coupled to an UHPLC 1290 Infinity-Series (Agilent Technologies, Waldbronn, Germany). As a column, an Eclipse Plus C18 B 1.5 *μ*m, 2.1 × 50 mm (Grace; Grace GmbH & Co KG, Worms, Germany) was used for separation. Samples of the same concentration were analyzed by linear gradient elution using H_2_O + 0.1% formic acid as solvent A and acetonitrile + 0.1% formic acid as solvent B. The gradient was from 5% to 100% solvent B in 6 min with a 2-min isocratic elution at 100% for solvent B.

### Bioassay-guided isolation of Pam for bioactivity assays

Small-scale purification was performed by a step-wise fractionation protocol. Bioactive fractions against *B. megaterium* XAD-16 fractions were submitted to Sephadex® LH-20 column (25 × 1000 mm, filled with 492 g Sephadex® LH-20 media, GE Healthcare, Uppsala, Sweden). The injection volume was 5 mL with a flow rate of 0.5 mL/min. 1.2 CVs were fractionated isocratically with 100% H_2_O with a fraction size of 2 mL. Antibiotically active fractions were pooled and dried in vacuo and purified subsequently by using a Reveleris® Flash Cartridge (Grace; Grace GmbH & Co KG, Worms, Germany) filled with 4 g C18 reversed phase resin coupled to a Reveleris® X2 Flash System (Grace; Grace GmbH & Co KG). The separation was accomplished by linear gradient elution using H_2_O + 0.1% formic acid as solvent A and methanol + 0.1% formic acid as solvent B. The gradient was from 3% to 15% solvent B in 20 min, followed by a 5-min gradient to 100% B ending at 100% B isocratic for 5 min at a flow rate of 18 mL/min.

Bioactive fractions were pooled, concentrated and purified subsequently by using a Grace HPLC column (250 × 20 mm) filled with 10 *μ*m GROM-Sil 120 ODS-5 ST coupled to an Agilent 1100 HPLC system (Agilent Technologies) with a multi wavelength ultraviolet detector (MWD). The separation was accomplished by linear gradient elution using H_2_O + 0.1% formic acid as solvent A and methanol + 0.1% formic acid as solvent B. The gradient was from 3% to 15% solvent B in 45 min, followed by a 5-min gradient to 100% B ending at 100% B isocratic for 5 min at a flow rate of 10 mL/min.

Subsequently, bioactive fractions were loaded onto a HPLC column (150 × 4.0 mm) filled with 5 *μ*m, 12 nm YMC-Pack C-8 (Octyl) (YMC Europe GmbH, Dinslaken, Germany) coupled to an Agilent 1100 HPLC system (Agilent Technologies) with a DAD UV detector. The samples were separated by linear gradient elution using H_2_O + 0.1% formic acid as solvent A and acetonitrile + 0.1% formic acid as solvent B at a flow rate of 1.5 mL/min. The gradient was from 3% to 20% for solvent B in 8 min with a 2-min hold at 20% for solvent B followed by a 2-min gradient to 100% B ending at 100% B isocratic for 5 min. Pam-containing fractions (mass spectrometric control) were used for bioactivity assays.

### High-resolution-LC-ESI-MS analytics

For the analysis of pure Pam an LTQ Orbitrap XL mass spectrometer (Thermo Scientific, Bremen, Germany) coupled to an Agilent 1260 HPLC system (Agilent Technologies) was used. A Grom-Sil ODS-4 HR 3 *μ*m, 50 × 2.0 mm column (Grace; Grace GmbH & Co KG) was used for separation. The sample was analyzed by linear gradient elution using H_2_O + 0.1% formic acid as solvent A and acetonitrile + 0.1% formic acid as solvent B. The elution started with an initial hold at 5% B for 1.6 min followed by an increase of solvent B to 100% in 7.9 min with a hold at 100% for 2.5 min.

### Cell toxicity assays

Cytotoxicity *in vitro* tests were performed using the commercial available cell line: BTI-Tn-5B1-4 cells (generous gift from Dr. Holger Hübner from the Friedrich-Alexander-Universität Erlangen-Nürnberg, Germany). The cells were maintained in 75 cm^2^ cell culture flasks (Roth) filled with 20 mL of serum-free Sf 900II medium (Lonza, Walkersville, MD, USA) and cultivated at 27°C in a cooling incubator. Cells were passaged once a week adjusting its initial concentration to 2E + 05 cells per mL using a counting chamber.

2E + 04 cells (100 *μ*L) were seeded in each well of a 96-well microtiter plate and incubated at 27°C for 24 h to allow cell adherence. Microtiter plates were centrifuged with 220*g* for 15 min and medium was removed and replaced with 100 *μ*L medium plus the different samples: Pam isolated from the supernatant of DSM25430 wt was resuspended (1:100) in 100 *μ*L SF 900 II medium with a 250 *μ*g mL^−1^ penicillin/streptomycin premix. Controls were performed as follows: SF 900 II medium served as a blank, HPLC solvent (1:100 diluted 5% acetonitrile + 0.1% formic acid) served as a negative control, and 10 *μ*L of lysis buffer (99.4 mL DMSO, 0.6 mL acetic acid and 10% SDS w/v) served as a positive control for cell toxicity. After 40 h of incubation at 33°C, plates were centrifuged (220 g for 15 min) and the medium was discarded. Subsequently, 100 *μ*L of fresh MTT (3-(4,5-dimethylthiazol-2-yl)-2,5-diphenyltetrazoliumbromid) solution (0.5 mg/mL in SF 900 II medium) was added to the cells and incubated at 27°C for 3 h. Microtiter plates were centrifuged again at 220*g* for 10 min and the supernatant was carefully aspirated without damaging the cell pellet. To disrupt the cells and liberate formazan, 100 *μ*L lysis buffer (99.4% DMSO mL, 0.6% acetic acid, 10% SDS w/v) were added and the plate was incubated for 5 min at 300 rpm on a vibrate platform shaker (Titramax 1000; Heidolph, Schwabach, Germany) at room temperature. The OD corresponding to cell-absorbed formazan, and thus to cell vitality after the treatment was analyzed with the SynergyHT ELISA reader (BioTek, Bad Friedrichshall, Germany) using 595 nm excitation wavelength.

### Infection assays

To analyze the functional role of Pam during pathogenesis, an exposure bioassay was performed as described by Genersch et al. (2005 and) Genersch et al. (2006). Preparation of spore suspension for infection assays was performed as previously described (Genersch et al. 2005, 2006; Rauch et al. 2009; Poppinga et al. 2012; Fünfhaus et al. 2013). Spore concentrations were calculated by plating out serial dilutions on CSA agar plates and counting colony forming units (CFUs). Spore suspensions with equal concentrations of CFUs were prepared for both wild-type and knock-out. Larvae of first instar were infected with *P. larvae* ERIC II wild-type (DSM25430) and the Pam deficient knock-out mutant (DSM25430 Δ*pam*A) by adding corresponding spores in a final concentration of 500 cfu/mL (about LC_100_) to the larval diet to ensure that infection of larvae was initiated by the same number of vegetative *P. larvae* for wild-type and knock-out bacteria. Control larvae were fed with normal larval diet, consisting of 66% royal jelly (v/v), 3.3% glucose (w/v) and 3.3% fructose (w/v). After 24 h, larvae were transferred to fresh food daily and monitored for 15 days. Dead larvae were removed, streaked out on CSA, incubated at 37°C for 2–3 days, and checked for AFB phenotypically and by PCR (Kilwinski et al. 2004). Only larvae that died of AFB infected by *P. larvae* ERIC II (DSM25430 wt) or by the Pam-deficient knock-out (DSM25430 Δ*pam*A) mutant, respectively, were considered for statistical calculations. Knock-out stability was proved by PCR with the gene-specific primer pair (Table1) PKSA_F and PKSA_R, flanking the intron insertion site 1080. PCR amplicons were analyzed by gel electrophoresis on a 1% agarose gel, stained with ethidium bromide and visualized by UV light.

Total mortality was depicted as mean values ± SD and statistically analyzed by Student's *t*-test. Survival curves were plotted and two-way ANOVA was used to calculate significant differences. Infection assays for DSM25430 wt, DSM25430 Δ*pam*A mutant and control larvae were carried out three times with 30 replicates each, statistical analysis was performed using GraphPad Prism 6 software.

## Results and Discussion

### Genomic organization of the Pam NRPS/PKS hybrid gene cluster

**Figure 2:**
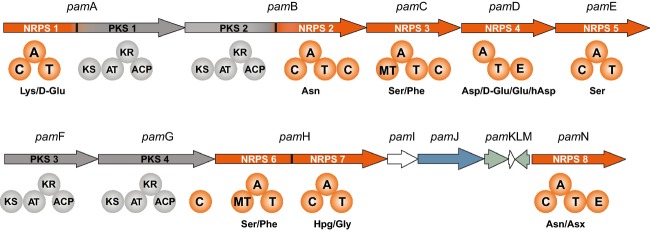
Domain organization derived from the pam secondary metabolite gene cluster in the genome of *Paenibacillus larvae* DSM25430. In the genome of *P. larvae* DSM25430 (NCBI Reference Sequence NC_023134.1) an NRPS/PKS hybrid gene cluster could be identified. The gene cluster of the putative NRP/PK hybrid paenilamicin (*pam* cluster) consisted of five NRPS, two PKS, and two NRPS/PKS hybrid genes. A, adenylation domain; C, condensation domain; T, thiolation domain; E, epimerization domain; MT, N-methyl transferase; KS, ketosynthetase; AT, acetyltransferase; KR, ketoreductase, and ACP, acyl carrier protein**.** Domain prediction was performed using SBSPKS (Anand et al. 2010). NRPSs, nonribosomal peptide synthetases; PKSs, polyketide synthases.

The complete genome of *Paenibacillus larvae* DSM25430 (ERIC II) has been sequenced (NCBI Reference Sequence NC_023134.1) and it has been shown to harbor several NRPS and NRPS/PKS hybrid clusters (Djukic et al. 2014). One of these clusters codes for a trimodular NRPS synthesising the tripeptide sevacidin (Garcia-Gonzalez et al. 2014). Further analysis now revealed a gene cluster coding for a mixed NRPS/PKS hybrid gene cluster termed *pam* cluster, with the compound Pam as its correspondent biosynthesis product. The genomic organization of the *pam* cluster revealed a size of ∼60 kb comprising five NRPS genes, two PKS genes, and two NRPS/PKS hybrid genes (gene locus tags ERIC2_c18040 – ERIC2 _c180170) (Fig.2, Table S1). The hybrid NRPS/PKS coding genes were assigned as *pam*A and *pam*B. Each of these hybrids contains an A domain (A1 and A2) for activation of corresponding amino acid substrates. The genes *pamC, pam*D, *pam*E, *pam*H, and *pam*N code for NRPSs whereas *pam*F and *pam*G code for PKSs.

BLAST analysis (Altschul et al. 1990, 1997) did not reveal any similarity to known NRPS/PKS gene clusters. In order to gain insight into the cognate amino acid substrates, we used several software tools and databases which allow predicting of substrate specificity of amino acid-activating adenylation domains (A domains) of NRPSs. NRPSpredictor2 predicts A-domain substrate specificity based on sequence and structural information about the active site of the domain (http://nrps.informatik.uni-tuebingen.de/) (Rausch et al. 2005; Röttig et al. 2011). This program recognized the A2, A5, and A8 domains as well conserved and predicted the activation of Asn (90%), Ser (100%), and Asn (100%), respectively (Table2). The other A domains of the *pam* cluster were less well conserved and, hence, provided only poor predictions: Lys (60%), Phe (50%), Asp (70%), Phe (50%), and Gly (70%) were predicted to be recruited by the A1, A3, A4, A6, and A7 domains, respectively (Table2). Modules NRPS4 and NRPS8 contain an additional epimerization domain for the stereochemical conversion of the activated l-amino acid into its d-amino acid enantiomer, suggesting d-Asp and d-Asn as constituents of Pam, respectively. Closer inspection of the four PKS modules revealed, next to a standard set of ketosynthase (KS), AT and ACP, that they each contains a KR domain, indicating the transformation of a *ß*-carbonyl into a *ß*-hydroxy group. The *pam*G gene codes for a PKS with a C-terminal C domain. While the predictor software was unable to predict the function of the second domain, a BLAST comparison of the domain sequence suggested an acyltransferase domain. We next used the NRPS-PKS tool of the SBSPKS software (http://www.nii.ac.in/∼pksdb/sbspks/master.html) which allows identifying various catalytic domains in the sequence of Type I PKS proteins and comparing them with experimentally characterized PKS/NRPS clusters cataloged in the backend databases of SBSPKS (Anand et al. 2010). Analysis of the *pam* gene cluster using this software predicted two additional domains in NRPS module 3 and NRPS module 6, suggesting an *N*-methylation of the incorporated amino acids, presumably Phe (Table2). Finally, we used NRPS/PKS analysis software (http://nrps.igs.umaryland.edu/nrps/2metdb), which has been designed for the prediction of microbial secondary metabolic pathways from DNA sequence data of gene clusters leading to the production of modular PKs and nonribosomally encoded peptides (Bachmann and Ravel 2009). Comparison of the results obtained by all three software tools (SBSPKS, NRPSpredictor2 and NRPS/PKS analysis) for the A domain specificity verified recruitment of Asn by A2 (NRPSpredictor 2 and SBSPKS), Ser by A5 (NRPSpredictor, SBSPKS, NRPS/PKS analysis), 4-hydroxyphenylglycine (Hpg) by A7 (PKS/NRPS analysis, SBSPKS), and Asn (SBSPKS, NRPSpredictor2) or Asx (PKS/NRPS analysis) by A8 (Table2). Between *pam*H and *pam*N, five genes (*pam*I, *pam*J, *pam*K, *pam*L, *pam*M) are located (Fig.2) that were analyzed by BLAST. *Pam*I and *pam*L could not be identified (hypothetical proteins) whereas *pam*J, *pam*K, and *pam*M were identified and annotated as cyclic peptide transporter, 3-hydroxy-CoA dehydrogenase, and transcription antiterminator-like protein NusG, respectively (Djukic et al. 2014).

**Table 2:** *In silico* prediction of A-domain specificity.

A domain	NRPSpredictor2	SBSPKS	PKS/NRPS analysis
1	Lys	d-Glu	NO HIT
2	Asn	Asn	NO HIT
3	Phe	Ser	NO HIT
4	Asp	d-Glu	Asp/hAsp/Glu
5	Ser	Ser	Ser
6	Phe	Ser	NO HIT
7	Gly	Hpg	Hpg
8	Asn	Asn	Asx

PKS, polyketide synthases; NRPS, nonribosomal peptide synthetases.

Recently, it was noted that the here described 3′-end of the Pam biosynthesis gene cluster comprising *pam*H to *pam*N and the xenocoumacin (XCN) biosynthesis gene cluster identified in *Xenorhabdus nematophila* contain homologous genes (Reimer et al. 2011). The *P. larvae* NRPS7 (PamH) was reported to recruit Hpg confirming our prediction (see above) and the NRPS PamN was shown to be homologous to the starting module XcnA of the XCN biosynthesis gene cluster (Reimer et al. 2011) as were corresponding modules in the biosynthesis gene clusters for amicoumacin (*B. pumilus*), zwittermicin (*B. thuringiensis*), colibactin (*E. coli*), and other similar NRPS/PKS clusters with unknown products (Reimer et al. 2011). All of these starting modules are predicted to be specific to Asn or Asx (Reimer et al. 2011). The XCN biosynthesis gene cluster also harbors the gene *xcn*G which was originally described as encoding a peptide transporter involved in the resistance mechanism (Reimer et al. 2009). However, most recent studies revealed that XcnG has a peptidase function which is necessary for prodrug activation (Reimer et al. 2011). The *X. nematophila* peptidase XcnG was shown to be a homolog of PamJ (Reimer et al. 2011) suggesting that *pam*J might be a peptidase gene rather than a peptide transporter gene. However, peptidase function of PamJ needs to be confirmed experimentally especially since considerable structural differences between XcnG and PamJ exist. Reimer and co-workers proposed two different domain architecture types for these prodrug-activating peptidases. Type I with XcnG as the model peptidase comprises a signal peptide followed by a peptidase domain and three C-terminal transmembrane helices. In contrast, *P. larvae* PamJ has type II domain architecture with nine transmembrane helices followed by an ABC transporter domain presumably located in the cytoplasm (Reimer et al. 2011). Therefore, an additional transport function of PamJ is likely but needs to be confirmed experimentally.

Xenocoumacins are produced by bacteria of the genus *Xenorhabdus*. These bacteria live in symbiosis with nematodes of the genus *Steinernema* which infect and kill insects. The proposed role of XCN in this symbiotic entomopathogenic complex is either to help killing the insect or to help killing the bacteria living inside the insect gut and competing with *Xenorhabdus* for food (Reimer et al. 2011). Interestingly, the same roles can be discussed for Pam. It might either act as an antibiotic killing bacterial competitors of *P. larvae* in the honey bee larval gut or it might act as a toxin helping *P. larvae* to kill the honey bee larvae. To address these questions, we functionally analyzed Pam.

### Paenilamicin is a nonribosomal peptide antibiotic

**Figure 3:**
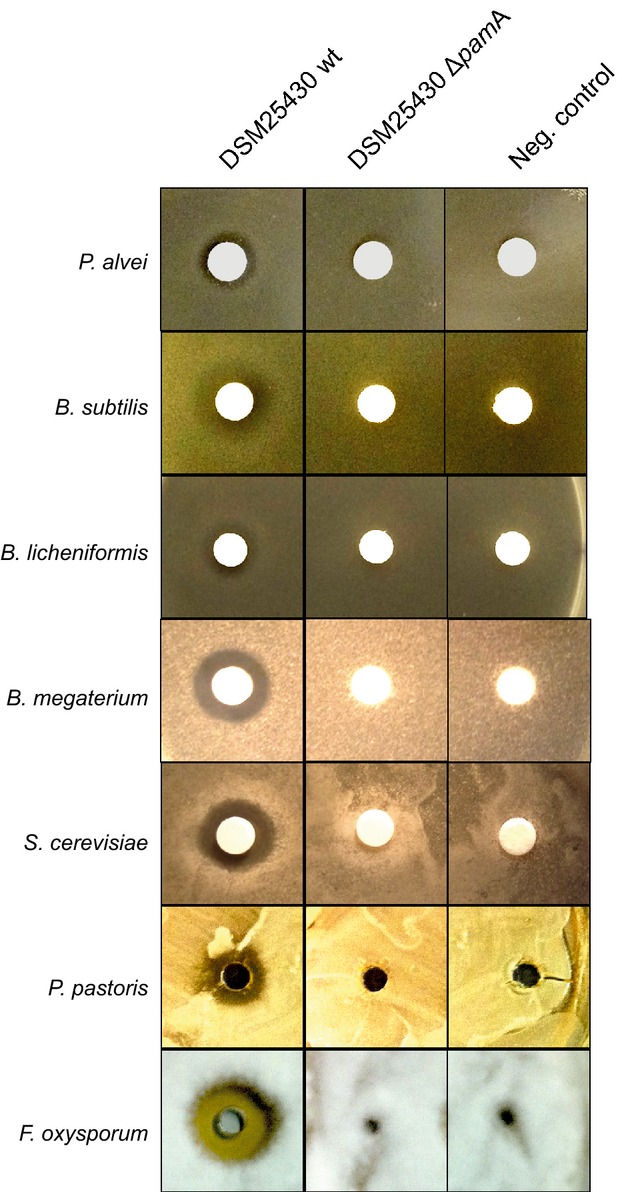
Contribution of paenilamicin to the antibacterial and antifungal activity of *P. larvae* culture supernatants. *P. larvae* ERIC II wild-type strain DSM25430 and mutant strain DSM25430 **Δ***pam*A lacking paenilamicin production were grown in MYPGP medium at 30°C. Activity of culture supernatants against different bacteria (*Paenibacillus alvei*, *Bacillus subtilis*, *B. licheniformis,* and *B. megaterium*) and fungi (*Saccharomyces cerevisiae*, *Pichia pastoris*, and *Fusarium oxysporum*) was tested by agar diffusion assays. Neg. control: MYPGP medium.

*Paenibacillus larvae* have been reported to produce antibacterial activity against different Gram-negative and Gram-positive bacteria including *B. subtilis* and *P. alvei* (Holst 1945). Nonribosomally synthesized peptide-polyketide hybrids like Pam often have antibiotic activity. In order to elucidate the contribution of the functional *pam* gene cluster and hence its biosynthetic product to the antibiotic activity of *P. larvae*, a knock-out mutant of *P. larvae* (DSM25430 Δ*pam*A) was generated, which was interrupted in the NRPS/PKS gene *pam*A. Subsequently, culture supernatants of wild-type *P. larvae* (DSM25430 wt) and mutant *P. larvae* lacking Pam production (DSM25430 Δ*pam*A) were tested for absence or presence of inhibitory activity against various bacteria (*P. alvei*, *B. subtilis*, *B. licheniformis*, and *B. megaterium*) and fungi (*Sacchoaromyces cerevisiae*, *Pichia pastoris*, and *Fusarium oxysporum*) selected because of their potential association with honey bees (Bailey 1963; Gilliam et al. 1974; Gilliam and Prest 1987; Snowdon and Cliver 1996; Gilliam 1997; Yoder et al. 2013). Culture supernatants of the wild-type *P. larvae* strain inhibited growth of all tested microorganisms although differences in inhibitory potential were evident (Fig.3). In contrast, culture supernatants of the DSM25430 Δ*pam*A mutant did not show any inhibitory activity. These results clearly linked integrity and thus functionality of the *pam* gene cluster to antibacterial/antifungal activity of *P. larvae* culture supernatants. So far we only tested some of the microorganisms *P. larvae* might encounter when infecting honey bee larvae. Once the larval microbiome is fully analyzed and, hence the potential competitors of *P. larvae* in the larval gut and the dead larvae are known, it will be of special interest to identify the target organism(s) of Pam. It will also be interesting to explore the potential of Pam in anti-infective therapy by elucidating the spectrum of medically relevant microorganisms inhibited by this antibiotic.

### Identification and purification of Pam

**Figure 4:**
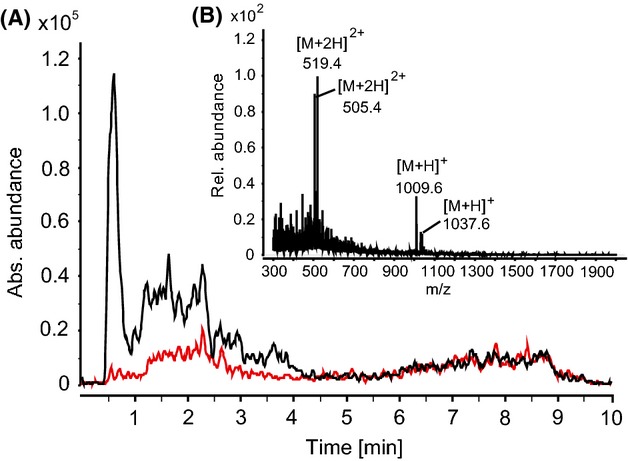
Identification of paenilamicin by HPLC-ESI-triple quadrupole MS from *P. larvae* secretome after sample workup. (A) Comparison of extracted ion chromatograms (EIC, *m/z* 519.0–519.5 corresponding to twofold charge state (*z* = 2) of paenilamicins) of a 90% XAD-16 fraction from wild-type strain DSM25430 (black) with mutant strain DSM25430 Δ*pam*A (red) (cultivation time 72 h). (B) Mass spectrum of a fraction (90% XAD-16) of *P. larvae* DSM25430 wild-type. Molecular masses of paenilamicins: [M+H]^+^ = 1009.6 Da and 1037.6 Da (elution time 0.397–0.843 min). No corresponding peaks were found for the mutant strain (data not shown). HPLC, high-pressure liquid chromatography; MS, mass spectrometry.

In order to further characterize the putative biosynthetic product of the *pam* gene cluster, a comparative metabolic profiling using HPLC mass spectrometry was employed. Direct HPLC-ESI-MS analysis of culture filtrates of *P. larvae* wild-type and *P. larvae* Δ*pam*A did not render any mass peaks which could be assigned to Pam or derivatives (Fig. S1). This finding could be attributed to signal suppression in mass spectrometry by high matrix background of the culture filtrate. Therefore, we performed a polarity-based fractionation of whole *P. larvae* DSM25430 Δ*pam*A supernatants and of the corresponding parent wild-type strain *P. larvae* DSM25430 by XAD-16 chromatography. Briefly, 50 mL of whole supernatants of the wild-type and the *pam*A knock-out were incubated with XAD-16 resins. Fractionation of the supernatants was accomplished with increasing concentrations of methanol (0–100%) as eluent. Subsequently, antibacterial activity of the eluted fractions was tested against *B. megaterium*. The DSM25430 wt 90% methanol fraction, that was highly active against *B. megaterium*, and the corresponding DSM25430 Δ*pam*A 90% fraction that showed no antibiotic activity were chosen for comparative metabolic profiling using reversed-phase HPLC electrospray ionization mass spectrometry (RP-LC-ESI-MS). Careful comparison of the obtained spectra indicated a compound eluting at R_t_ = 0.6 min that was absent in the corresponding fraction of the mutant strain (Fig.4A). By HPLC-ESI mass spectrometry two singly-charged ion peaks with molecular masses of [M+H]^+^ = 1009.6 and 1037.6 Da could be identified in the 90% methanol fractions of the *P. larvae* wild-type strain that were absent in the 90% methanol fractions of the DSM25430 Δ*pam*A strain (Fig.4B). Due to the gene inactivation experiments of the *pam* gene cluster we could unambiguously assign these peaks as products of the NRPS/PKS machinery that is encoded by the *pam* gene cluster.

**Figure 5:**
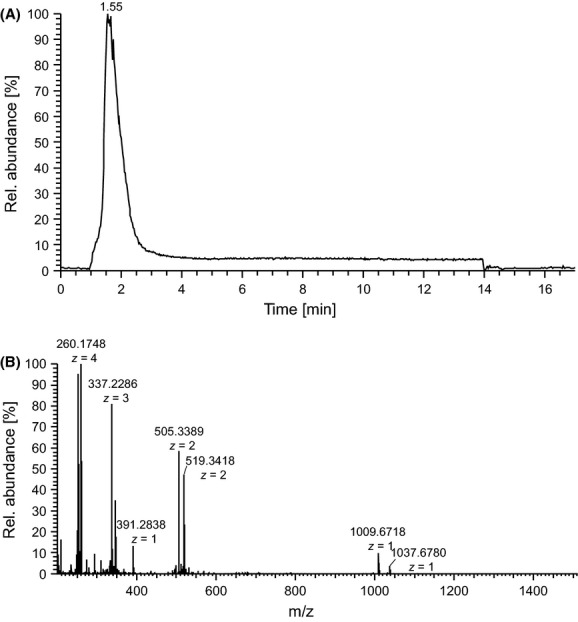
HR-MS analysis of purified paenilamicin. (A) Total ion chromatogram (TIC) of purified paenilamicin. (B) Corresponding HR mass spectrum (1.26–2.37 min) showing both paenilamicin derivatives with different charge states. *m/z* 391.2838 is a compound also present in blank measurements. HR, high resolution; MS, mass spectrometry.

To verify our assumption that the identified peaks are responsible for the antibiotic activity, we performed a bioactivity-guided small-scale purification from 1 L culture of *P. larvae* DSM25430 wt by a stepwise fractionation protocol with *B. megaterium* as the indicator strain (see Materials and Methods). Fractions containing Pam were collected from the semi-preparative C8 HPLC separation and pooled. High sample purity was confirmed by a single peak in the chromatogram (MS detection) and high-quality MS data with little background (Fig.5A and B). Subsequent high-resolving HPLC-ESI Orbitrap-MS of the pooled fractions showed that Pam consists of two components ([M+H]^+^ = 1009.6718 and 1037.6780 Da; Fig.5B). Thus, the purified Pam mixture (∼1.2 mg) was used for further functional assays to characterizing bioactivities.

### Purified Pam has antibacterial, antifungal, and cytotoxic activity

**Figure 6:**
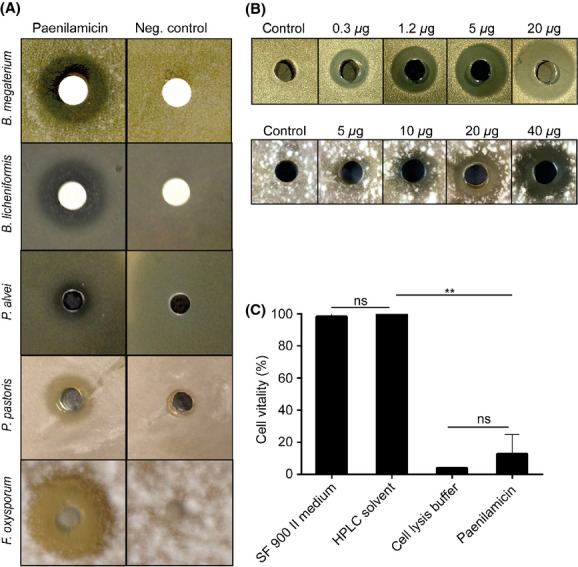
Antibacterial, antifungal, and cytotoxic activity of purified paenilamicin. (A) Isolated paenilamicin (left column) showed antibiotic activity against *B. megaterium, B. licheniformis, P. alvei, P. pastoris,* and *F. oxyporum*. HPLC solvent served as negative control. (B) Increasing concentrations of purified paenilamicin were tested in agar diffusion assays against *B. megaterium* (0.3–20.0 *μ*g Pam) and *F. oxysporum* (5.0–40.0 *μ*g Pam) as bacterial and fungal indicator strain, respectively. Water was used as a control. (C) Tn5 cells were incubated for 40 h in cell culture medium (SF900 II) or in cell culture medium supplemented with HPLC solvent as negative control, cell lysis buffer as positive control, and paenilamicin isolated from DSM25430 wt. Quantitative measure of cell vitality obtained by the MTT test is shown in the bar graph. Cells incubated with paenilamicin showed a significantly decreased vitality compared to the two negative controls. Bars represent mean values ± SD of three replicates, analyzed by Student's *t*-test (**P < 0.01). HPLC, high-pressure liquid chromatography.

To further confirm the results obtained with *P. larvae* culture supernatants, we used purified Pam in agar diffusion assays performed with *B. megaterium, B. licheniformis, P. alvei, P. pastoris*, and *F. oxysporum* as bacterial and fungal indicator strains, respectively. Halos around the disk impregnated with 20 *μ*L Pam (*B. megaterium* and *B. licheniformis*) or the hole containing 40 *μ*L Pam (*P. alvei, P. pastoris*, and *F. oxysporum*) confirmed the results obtained with culture supernatants and further substantiated that Pam has antibacterial and antifungal activity (Fig.6A). This activity was concentration dependent as revealed by an increased inhibition of *B. megaterium* and *F. oxysporum* when increasing concentrations of purified Pam (0.3–20.0 and 5.0–40.0 *μ*g, respectively) were tested (Fig.6B). Some secondary peptide metabolites also have cytotoxic activity. In order to further converge to the *in vivo* function of Pam we, therefore, investigated its putative cytotoxicity on insect cells by using the insect cell line Tn5 derived from *Trichoplusia ni* (Lepidotera). Cell vitality was determined via MTT test for Tn5 cells incubated in cell culture medium alone (SF 900 II medium) or in cell culture medium supplemented with HPLC solvent (1:100 diluted 5% acetonitrile + 0.1% formic acid) as negative control, with cell lysis buffer as positive control or with Pam (Fig.6C). While cell vitality was unaltered in the presence of HPLC solvent (100 ± 10.51% vital cells; *P* = 0.89), incubation with Pam led to a significant decrease in cell vitality (12.86 ± 11.91% vital cells; *P* = 0.0024, Student's *t*-test) after 40 h comparable to the effect of cell lysis buffer (4.01 ± 0.22% vital cells; *P* = 0.0008, Student's *t*-test). These results suggested that in addition to its antibacterial and antifungal activity, Pam also is cytotoxic to insect cells.

Biosynthesis gene clusters containing genes coding for homologs of XcnA and XcnG are described to be evolutionary conserved (Reimer et al. 2011). Among these clusters is the biosynthesis gene cluster leading to the production of colibactin (Homburg et al. 2007; Reimer et al. 2011). The polyketide-peptide hybrid colibactin, produced by strains of *Escherichia coli* harboring a genomic island called “pks,” has been described as cytotoxin or, more precisely, as genotoxin (Nougayrede et al. 2006; Cuevas-Ramosa et al. 2010). However, insect cells intoxicated by Pam did not show the cytopathic effect described for colibactin, i.e., megalocytosis, characterized by a progressive enlargement of the cell body and nucleus and the absence of mitosis, indicating that cytotoxicity of Pam is different from colibactin's effect on eukaryotic cells.

### Paenilamicin is involved in the time course of infection

**Figure 7:**
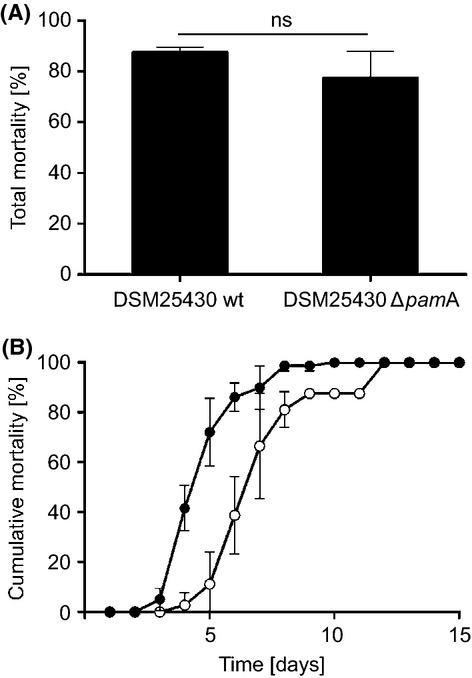
Role of paenilamicin during infection of honey bee larvae. (A) First instar honey bee larvae were infected with *P. larvae* DSM25430 wt and DSM25430 Δ*pam*A. Daily mortality due to *P. larvae* infection was recorded and total mortality was calculated. Bars represent the percentage of exposed larvae that died from American Foulbrood after 15 days. Bars represent mean values ± SD of three independent exposure bioassays each with three groups (control, wt and mutant bacteria) and 30 larvae per group. Data were analyzed by Student′s *t*-test. No significant difference in total mortality was observed. (B) Daily mortality due to *P. larvae* infection was recorded and cumulative mortality was calculated. Larvae infected with DSM25430 Δ*pam*A died as fast as larvae that had been infected with wild-type bacteria, however, onset of larval dying was delayed resulting in a significant parallel shift of the cumulative mortality curve to the right. Curves represent mean cumulative mortality ± SD for each day of three replicas with 30 larvae each. Data were analyzed by two-way ANOVA (*P* = 0.028).

Based on the *in vitro* cytotoxic activity of purified Pam against insect cells we hypothesized that Pam might act as toxin during *P. larvae* infection. To test this assumption we performed exposure bioassays with wild-type *P. larvae* (DSM25430 wt) and the mutant strain lacking Pam production (DSM25430 Δ*pam*A). Surprisingly, total mortality did not differ significantly between the groups infected with wild-type or mutant bacteria (Fig.7A). This result did not corroborate a function of Pam as toxin during infection despite its cytotoxicity *in vitro* and exemplified that in the *P. larvae*/honey bee larvae system it is obviously not always possible to infer from *in vitro* data on *in vivo* function.

Analyzing the time course of infection revealed that the curve for cumulative mortality was shifted to the right for the groups infected with the mutant strain (Fig.7B) indicating that in these groups the onset of larval dying was significantly delayed in comparison to the groups infected with wild-type bacteria (*P* = 0.028; two-way ANOVA). Accordingly, the LT_100_ also significantly differed between both strains (Student's *t*-test, *P* = 0.0142). The wild-type strain DSM25430 needed 8.33 ± 1.52 days to kill all infected larvae, whereas it took the mutant strain DSM25430 Δ*pam*A 12.0 ± 0 days to accomplish killing all infected animals. These results suggested that Pam although not influencing total mortality influenced the time course of disease and, hence, can be considered a virulence factor.

These assays were all performed with *P. larvae* DSM25430, a strain belonging to the genotype ERIC II, and a mutant thereof. So far, no toxin genes could be identified in this genotype (Fünfhaus et al. 2009; Djukic et al. 2014) while two functional AB toxins, Plx1 and Plx2, have been characterized in *P. larvae* ERIC I quite recently (Fünfhaus et al. 2013). Gene disruption in these toxin genes led to a significant reduction in mortality indicating that they are virulence factors and play an important role during pathogenesis (Fünfhaus et al. 2013). If the function of Pam were to act as cytotoxin during infection and to help breaching the epithelial cell layer (Yue et al. 2008) we would have expected a similar significant reduction in total mortality in the absence of this toxin, especially because so far no toxin gene has been identified in this strain (Djukic et al. 2014). Instead, absence or presence of Pam production did not make a difference in total mortality but instead a delay in the onset of larval dying was observed in those larvae that were infected with the mutant bacteria. This suggested that Pam might play a role in the very early phase of infection when *P. larvae* germinates and starts to colonize the larval midgut. However, the observed effect caused by the absence of Pam cannot simply be explained by differences in germination efficiency between the wild-type and the mutant strains. Both, germination and growth of the mutant strain were not negatively influenced in cultured *P. larvae* when compared to the wild-type strain (Fig. 1) and all larvae were infected with the same concentration of CFUs, hence, with the same concentration of germinating spores. Therefore, more experimental work is necessary to unravel the exact function of Pam in pathogenesis of *P. larvae* infections and to explain its influence on virulence of *P. larvae*.

In summary, we here presented the identification, isolation, and biological characterization of Pam, a peptide-polyketide hybrid produced by the bacterial honey bee pathogen *P. larvae*. We were able to prove that Pam which consists of two components with molecular masses of [M+H]^+^ = 1009.6718 and 1037.6780 Da has antibacterial and antifungal activity. In addition, we demonstrated that paenilamcin has cytotoxic activity against insect cells, although a function as toxin during *P. larvae* infection of honey bee larvae could not be confirmed. However, in larvae infected by *P. larvae* lacking Pam production a delayed onset of larval mortality by unaltered total mortality could be observed in comparison to larvae infected by wild-type bacteria. These results suggested that Pam is involved in determining the time course of disease and, hence, can be considered a virulence factor of *P. larvae*. Further experimental work is necessary to elucidate the chemical structures of Pam as well as mechanistic details of Pam activity during AFB pathogenesis which will finally unravel the full potential of this secondary metabolite.

## Supporting Information

**Figure S1.** of LC-ESI-Orbitrap-MS profiling of metabolites present in the crude supernatant. (A) Total ion chromatogram (TIC) of *P. larvae* DSM25430 wt and (B) total ion chromatogram (TIC) of *P. larvae* DSM25430 Δ*pam*A. Paenilamicin could not be detected directly from crude supernatants due to early elution in the dead volume and ion suppression. The secondary metabolite sevadicin is indicated with a red arrow. **Figure S2.** MS/MS fragmentation studies on paenilamicin by HPLC-ESI-triple quadrupole MS from *P. larvae* secretome after sample workup. (A) MS/MS fragmentation spectrum with [M + 2H]^2+^ = 505.4 as precursor ion (♦). (B) MS/MS fragmentation spectrum with [M + 2H]^2+^ = 519.4 as precursor ion (♦). **Figure S3.** HPLC UV analytics of pure paenilamicin. (A) Chromatogram of pure paenilamicin monitored at *λ* = 214 nm. (B) Chromatogram of a blank measurement (H_2_O) monitored at *λ* = 214 nm. Paenilamicin shows practically no retention at the C18 column. **Table S1.** Homology analysis of the *pam* gene cluster products.
