# Supplementary material for: Biological effects of paenilamicin, a secondary metabolite antibiotic produced by the honey bee pathogenic bacterium Paenibacillus larvae
Source: Microbiologyopen. 2014 Jul 16;3(5):642–56. doi: 10.1002/mbo3.195 (PMC4234257; doi:10.1002/mbo3.195)
Supplement: Supplementary file 1 — Figure S1. of LC-ESI-Orbitrap-MS profiling of metabolites present in the crude supernatant. (A) Total ion chromatogram (TIC) of P. larvae DSM25430 wt and (B) total ion chromatogram (TIC) of P. larvae DSM25430 ΔpamA. Paenilamicin could not be detected directly from crude supernatants due to early elution in the dead volume and ion suppression. The secondary metabolite sevadicin is indicated with a red arrow. Figure S2. MS/MS fragmentation studies on paenilamicin by HPLC-ESI-triple quadrupole MS from P. larvae secretome after sample workup. (A) MS/MS fragmentation spectrum with [M + 2H]2+ = 505.4 as precursor ion (♦). (B) MS/MS fragmentation spectrum with [M + 2H]2+ = 519.4 as precursor ion (♦). Figure S3. HPLC UV analytics of pure paenilamicin. (A) Chromatogram of pure paenilamicin monitored at λ = 214 nm. (B) Chromatogram of a blank measurement (H2O) monitored at λ = 214 nm. Paenilamicin shows practically no retention at the C18 column. Table S1. Homology analysis of the pam gene cluster products. [file mbo30003-0642-sd1.docx]

**Biological effects of paenilamicin, a secondary metabolite antibiotic produced by the honey bee pathogenic bacterium *Paenibacillus larvae***

Eva Garcia-Gonzalez^1,§^, Sebastian Müller^2,§^, Gillian Hertlein^1^, Nina Heid^1^, Roderich D. Süssmuth^2^, Elke Genersch^1,3, *^

^1^ Institute for Bee Research, Department of Molecular Microbiology and Bee Diseases, Friedrich-Engels-Str. 32, 16540 Hohen Neuendorf, Germany

^2^ Technische Universität Berlin, Institut für Chemie, 10623 Berlin, Germany

^3^ Freie Universität Berlin, Institute of Microbiology and Epizootics, Robert-von-Ostertag-Str. 7-13, 14163 Berlin, Germany

^§^Both authors contributed equally to this work

* Correspondence and reprints: E-mail: [elke.genersch@rz.hu-berlin.de](mailto:elke.genersch@rz.hu-berlin.de)

Tel.: ++49 (0)3303 293833

Fax: ++49 (0)3303 293840

Running head: *P. larvae* nonribosomal polyketide-peptide hybrid.

Key words: *Paenibacillus larvae*, paenilamicin, nonribosomal peptide-polyketide hybrid, antibacterial activity, antifungal activity, cytotoxic activity


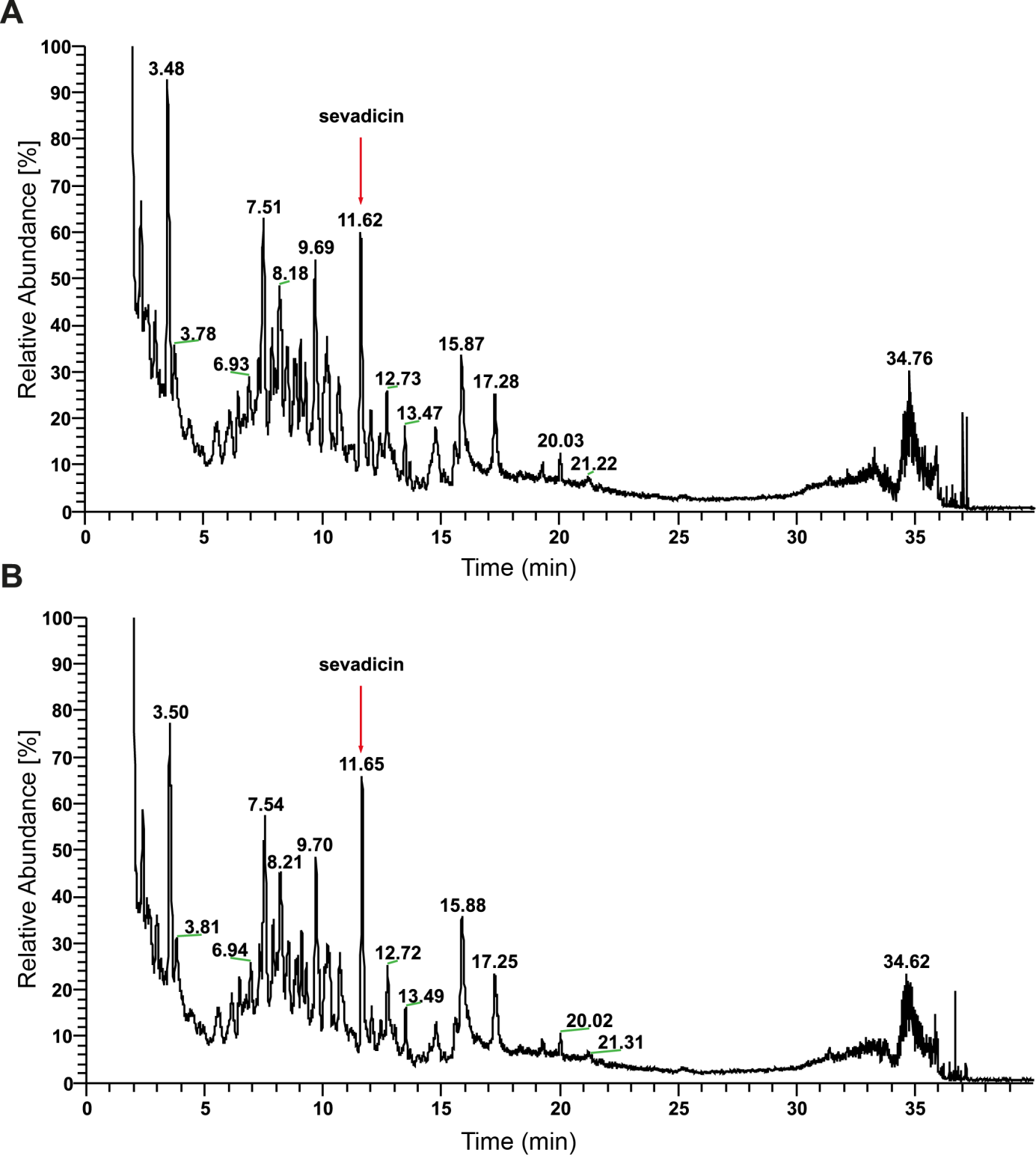


**Figure S1: Comparison of LC-ESI-Orbitrap-MS profiling of metabolites present in the crude supernatant.** A) Total ion chromatogram (TIC) of *P. larvae* DSM25430 wt and B) total ion chromatogram (TIC) of *P. larvae* DSM25430 ∆*plm*A. Paenilamicin could not be detected directly from crude supernatants due to early elution in the dead volume and ion suppression. The secondary metabolite sevadicin is indicated with a red arrow.


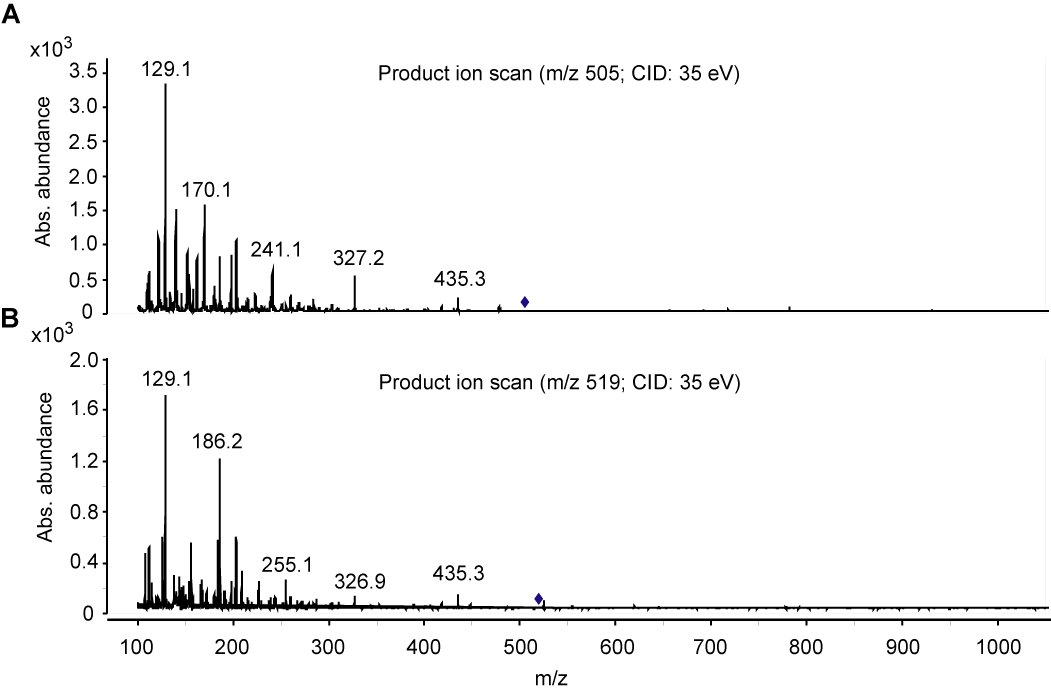


**Figure S2:** **MS/MS fragmentation studies on paenilamicin by HPLC-ESI-triple quadrupole MS from *P. larvae* secretome after sample workup.** A) MS/MS fragmentation spectrum with [M+2H]^2+^ = 505.4 as precursor ion ( ). B) MS/MS fragmentation spectrum with [M+2H]^2+^ = 519.4 as precursor ion ( ).


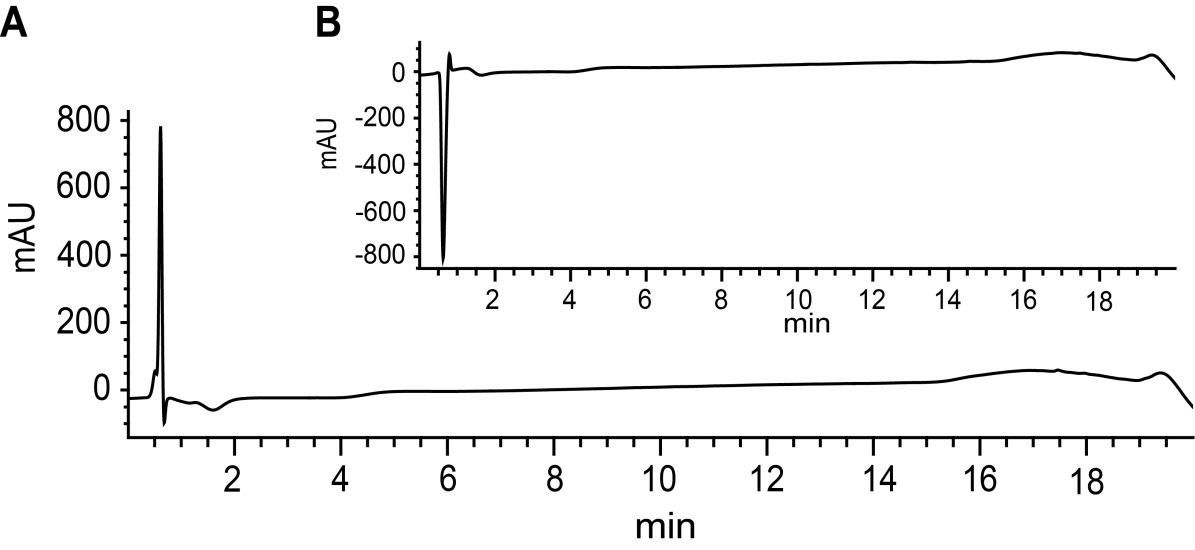


**Figure S3: HPLC UV analytics of pure paenilamicin.** A) Chromatogram of pure paenilamicin monitored at λ = 214 nm. B) Chromatogram of a blank measurement (H_2_O) monitored at λ = 214 nm. Paenilamicin shows practically no retention at the C18 column.

**Table S1:** Homology analysis of the *pam* gene cluster products

| **Protein** | **Organism** | **Identities/positives** | **Accession number** | **Proposed function/ homolog** |
| --- | --- | --- | --- | --- |
| PamA | *Brevibacillus laterosporus* | 45/65 | WP_018670478.1 | Hypothetical protein |
| PamB | *Clostridium papyrosolvens* | 40/60 | WP_020815481.1 | Beta-ketoacyl synthase |
| PamC | *Clostridium papyrosolvens* | 38/58 | WP_020816431.1 | AMP-binding protein |
| PamD | *Clostridium botulinum* | 41/62 | YP_002802629.1 | Amino acid adenylation-containing protein |
| PamE | *Paenibacillus peoriae* | 52/69 | WP_010349581.1 | NRPS (FusA) |
| PamF | *Paenibacillus dendritiformis* | 45/63 | WP_006675307.1 | Polyketide synthase subunit |
| PamG | *Cylindrospermum stagnale* | 41/58 | YP_007317768.1 | Beta-ketoacyl synthase family protein |
| PamH | *Clostridium papyrosolvens* | 38/59 | WP_020816431.1 | AMP-binding protein |
| PamI | *Paenibacillus alvei* | 24/41 | WP_021261934.1 | Hypothetical protein |
| PamJ | *Bacillus weihenstephanensis* | 47/69 | YP_001644645.1 | Cyclic peptide transporter |
| PamK | *Clostridium sp.* BNL1100 | 63/78 | YP_005146944.1 | 3-hydroxyacyl-CoA-dehydrogenase |
| PamL | *Clostridium termitidis* | 41/64 | WP_004626405.1 | Phosphopantetheine attachment site |
| PamM | *Brevibacillus laterosporus* | 51/68 | WP_003343549.1 | Transcription antiterminator |
| PamN | *Paenibacillus sp. OSY-SE* | 66/79 | WP_019424892.1 | Thioester reductase |

**HR-LC-ESI-MS analytics**

For the comparison of crude supernatants a LTQ Orbitrap XL mass spectrometer (Thermo Scientific, Bremen, Germany) coupled to an Agilent 1260 HPLC system (Agilent Technologies, Waldbronn, Germany) was used. For the comparison of the supernatants of *P. larvae* DSM25430 wt and *P. larvae* DSM25430 ∆*plm*A a Thermo Hypersil GOLD 5 µm, 150 mm × 2.1 mm column (Thermo Scientific, Bremen, Germany) was used for separation. The samples were analyzed by linear gradient elution using H_2_O + 0.1% formic acid as solvent A and acetonitrile + 0.1% formic acid as solvent B. For the analyses of crude supernatants the elution started with an initial hold at 5% B for 1 min, followed by an increase of solvent B to 40% in 19 min. The gradient ended with an increase of solvent B to 100% in 10 min with an isocratic hold at 100% for 5 min.

**LC-ESI-MS/MS analytics**

LC-ESI-MS/MS analytics were performed using an Agilent6410 Triple Quadrupole LC/MS system in positive ionization mode coupled to an UHPLC 1290 Infinity-Series (Agilent Technologies, Waldbronn, Germany). A VisionHT C18 Column 50 mm x 2 mm, 1.5 μm (Grace, Grace GmbH & Co KG, Worms, Germany) was used for separation. Samples were analyzed by linear gradient elution using H_2_O + 0.1% formic acid as solvent A and acetonitrile + 0.1% formic acid as solvent B. The gradient was from 5% to 100% solvent B in 6 min with a 2 min isocratic elution at 100% for solvent B. This method was used for product ion scans (collision energy: 35 eV (collision-induced dissociation, CID)) with paenilamicin ([M+2H]^2+^ = 505.4 or 519.4 Da) as precursor ions.

**Analytical HPLC screening**

The purified paenilamicin was screened using an Agilent 1100 HPLC system (Agilent Technologies, Waldbronn, Germany) with a DAD UV detector. 20 µl of the paenilamicin fraction was injected onto a HPLC column (50 × 2 mm) filled with 3 µm GromSil 120 ODS-5 ST (Grace, Grace GmbH & Co KG, Worms, Germany). The samples were analyzed by linear gradient elution using H_2_O + 0.1 % formic acid as solvent A and acetonitrile + 0.1 % formic acid as solvent B at a flow rate of 0.3 ml/min. The gradient was from 1 % to 30 % solvent B in 12 min, followed by an increase of solvent B to 100% in 4 min with a 3 min isocratic elution at 100 % for solvent B.
